# Platelets and cancer angiogenesis nexus

**DOI:** 10.1007/s10555-017-9673-1

**Published:** 2017-07-05

**Authors:** Marek Z. Wojtukiewicz, Ewa Sierko, Dominika Hempel, Stephanie C. Tucker, Kenneth V. Honn

**Affiliations:** 1 Department of Oncology, Medical University, 12 Ogrodowa St., 15-027, Bialystok, Poland; 2 Department of Clinical Oncology, Comprehensive Cancer Center, Bialystok, Poland; 3 Department of Radiotherapy, Comprehensive Cancer Center, Bialystok, Poland; 4 Bioactive Lipids Research Program, Department of Pathology-School of Medicine, Detroit, MI USA; 5 Department of Chemistry, Wayne State University, Detroit, MI USA; 6 Department of Oncology, Karmanos Cancer Institute, Detroit, MI USA

**Keywords:** Platelets, Angiogenesis, Cancer, Microparticles, Growth factors, MicroRNA, miRNAs

## Abstract

There has been remarkable insight into the importance of platelets in a wide range of pathophysiologic events, including inflammation and cancer progression. Thrombocytosis in cancer patients is a common finding. Tumor cells induce platelet activation and subsequent aggregation through direct and indirect mechanisms. Platelets are recognized to contribute to metastatic dissemination. There is plenty of evidence that components of the hemostatic system contribute to the process of angiogenesis. Furthermore, there are accumulated data on the substantial influence of blood platelets in the process of blood vessel formation during malignancy. Platelets appear to be the main physiologic transporters of proangiogenic and antiangiogenic factors. Moreover, they influence the process of angiogenesis through platelet-derived microparticles, microRNA, lipids, and variety of surface receptors. Platelets contribute to early and late stages of angiogenesis. Available data support the overall stimulatory effect of platelets on tumor angiogenesis. It raises the possibility that interfering with platelet function may be an effective antineoplastic treatment strategy.

## Introduction

Platelets, discovered by G. Bizzozero in 1882, were primarily recognized to play a role in hemostasis and thrombosis [1–3]. The presence of a thrombus accompanying cancer cells was observed over 130 years ago, and trombophilic state, both subclinical and clinically overt, is a frequent finding in cancer patients [3–7]. Thromboembolic complications may precede the diagnosis of cancer (“occult cancer”), occur during the natural course of neoplastic progression or be a complication of an oncologic treatment [8, 9]. Thrombocytosis, a paraneoplastic syndrome, frequently accompanies cancer growth and metastatic dissemination. It is observed in as many as 10–57% of cancer patients [6–10]. High platelet count correlates with poor prognosis in patients with colon, lung, gastric, renal, prostatic, cervical, endometrial, and ovarian cancer and in malignant mesothelioma [11–19]. Pretreatment thrombocytosis is a poor prognostic factor as well and reflects aggressive tumor behavior in malignant mesothelioma, cervical, colon, and non-small cell lung cancer patients [20, 21]. On the contrary, in pancreatic cancer patients, thrombocytopenia was associated with worse prognosis, thus indicating varied influence of platelets dependent on the type of cancer [22]. There is increased platelet turnover in cancer patients compared to healthy individuals, which corrects itself after anticancer treatment [23].

There has been remarkable insight into the importance of platelets in a wide range of pathophysiologic events, including inflammation and cancer progression. Platelets are recognized to contribute to metastatic dissemination [24, 25]. In experimental mouse models, inducing thrombocytopenia was associated with a diminished rate of metastases [26], whereas injection of human platelets to thrombocytopenic mice resulted in increased number of metastases *in vivo* [26, 27].

Formation of new blood vessels from pre-existing ones is a prerequisite of primary tumor growth, cancer cell intravasation, extravasation, and growth of cancer foci at distant sites [28–32]. It is widely known that angiogenesis is a rate-limiting process in cancer progression [29, 30]. There is plenty of evidence that components of the hemostatic system contribute to the process of angiogenesis [31–37]. Furthermore, there are accumulated data on the substantial influence of blood platelets in the process of blood vessel formation during malignancy [33]. Platelets appear to be the main physiologic transporters of the most important proangiogenic factor, vascular endothelial growth factor (VEGF), which implies their contribution to cancer angiogenesis as well [38]. The above hypothesis is supported by evidence that platelets are activated in tumor vasculature, which enables them to secrete their releasate directly within malignant tissue where they release VEGF upon activation [39–41]. Moreover, platelets stimulate capillary growth *in vitro* and angiogenesis *in vivo* [42, 43].

## Platelet structure and function

Platelets are the smallest as well as the most numerous morphologic blood elements (2 × 10^8^/mL) and are characterized by a short turnover time (5 to 7 days). These anucleated blood constituents are surrounded by a phospholipid membrane. The outer platelet membrane is composed of glycoproteins and is enriched with integrins that contribute to the adhesive and aggregative processes. Among integrins, the most important are glycoproteins: Ib-IX-V (GP Ib-IX-V), VI (GP VI), and IIb-IIIa (GP IIb-IIIa, also known as integrin αIIbβ3) [44–46]. Additional receptors in the platelet membrane include are protease-activated receptors (PAR-1—responsible for majority of thrombin activity—and PAR-4) [47], adhesion molecules from immunoglobulin and selectin families [44], as well as purinergic P2 receptors for nucleotides (adenosine diphosphate (ADT) and adenosine triphosphate (ATP)) [48]. Endothelial cell damage or alteration leads to exposure of the subendothelial extracellular matrix (ECM) components that are ligands for platelet adhesion and include various types of collagen, von Willebrand factor (vWF), laminin, vitronectin, proteoglycans, thrombosposndin, and fibronectin [49]. At high shear stress, collagen and collagen-bound vWF are important for platelet adhesion and activation [50]. The latter reversibly interacts with platelet GP Ib-IX-V resulting in reduced platelet velocity and platelet rolling over the collagen surface. This glycoprotein also facilitates platelet-endothelial cell (EC) adhesion (through P-selectin) and platelet-leukocyte adhesion (through Mac-1) [44]. In addition, vWF binds to platelet GPIIb-IIIa producing a bridge between platelets and collagen [51]. Firm adhesion of platelets to collagen is directly mediated by GPVI and GPIa-IIb [49], which, in turn, induces their activation and increases cytosolic calcium concentration. At low shear stress, GPVI binding to collagen sufficiently mediates adhesion and activation of platelets [44, 45]. During platelet activation, phosphatidylserine (PS) is exposed on the outer side of the membrane and microvesicles enriched with PS facilitate procoagulant activity [52]. Furthermore, upon activation, platelet membranes form many invaginations that extend their active surface. The cytoskeleton of the platelet is composed of numerous cross-linked elements, mainly actin, that connects with the cytoplasmic domain of GPIb-IX complexes as well as GPIa-IIa complexes [53]. Under physiologic conditions, the shape of platelets is discoid. However, upon activation, actin polymerizes and its subunits are rapidly reassembled into a variety of new structures such as filopodia and lamellipodia to dramatically generate new platelet shapes (balloon-like) depending on the external forces, extracellular signals, and physiologic requirements [54, 55]. Once activated, platelets form platelet-derived microparticles (PMPs) and exosomes and provide a source of nucleic acids as well.

Platelets are enriched in three types of specific granules (α-granules, dense granules, and lysosomes) that store a diverse array of products, as well as mitochondria and a dense tubular system that facilitates delivery of energy and biochemical messengers that contribute to platelet reactivity [56]. Alpha granules (50–80 per human platelet) are most numerous and store large proteins that play a role in adhesion and aggregation. Dense granules (three to eight per human platelet) are enriched with small non-protein molecules and have far fewer factors that upon secretion facilitate recruitment of other platelets [56, 57]. Lysosome function is not well characterized, but they are loaded with hydrolases that participate in the elimination of platelet aggregates [56, 57] (Table 1).

**Table 1:** Platelet releasate from three major forms of storage granule products: α-granules, dense granules, and lysosomes [33, 57]

Platelet granule	Constituents
α-Granules	Adhesion molecules (e.g., vWF, αIIbβ3, αvβ3, P-selectin, thrombospondin, fibrinogen, fibronectin)
	Coagulation factors (prothrombin, fibrinogen, factor V, factor VIII)
	Fibrinolytic factors (α2-macroglobulin, plasminogen, PAI-1, SERPINE1, uPA)
	Growth factors (VEGF-A, VEGF-C, PDGF, bFGF, EGF, HGF, IGF1, TGFβ)
	Proagiogenic and antiagiogenic factors (angiopoietin-1, angiostatin, S1P)
	Tissue remodeling matrix metalloproteinases (MMP-1, MMP-2, MMP-3, MMP-9, MT1-MMP)
	Tissue inhibitor of metalloproteinases (TIMPs: TIMP-1, TIMP-2, TIMP-4)
	Disintegrin
	Metalloproteinases (ADAMs: ADAM-10, ADAM-17, ADAMTS-13)
	Proinflammatory mediators (CXCL1 (GRO-α), CXCL4 (PF4), CXCL5 (ENA-78), CXCL7 (PBP, β-TG, CTAP-III, NAP-2), CXCL8 (IL-8), CXCL12 (SDF-1α), CCL2 (MCP-1), CCL3 (MIP-1α), CCL5 (RANTES), CCL7 (MCP-3), CCL17 (TARC), PAF, acetylhydrolase, LPA)
	Immunologic molecules (C1 inhibitor, IgG)
	Other proteins (albumin, α1-antitrypsin, Gas6, HMWK
Dense granules	Ions (calcium, magnesium, phosphate, and pyrophosphate)
	Nucleotides (ATP, GTP, ADP, GDP)
	Membrane proteins (tetraspanins, LAMP2)
	Transmiters (5-HT, epinephrine, histamine)
	Protease inhibitors (TFPI)
Lysosome	Phospholipase A protease glycohydrolase enzymes

*vWF* von Willabrand factor, *αIIbβ3* glycoprotein IIb-IIa, *PAI-1* plasminogen activator inhibitor-1, *uPA* urokinase plasminogen activator, *VEGF-A* and *VEGF-C* vascular endothelial growth factor A and C, *PDGF* platelet-derived growth factor, *bFGF* basic fibroblast growth factor, *EGF* epidermal growth factor, *HGF* hepatocyte growth factor, *IGF1* insulin-like growth factor 1, *TGFβ* transforming growth factor β, *S1P* sphingosine-1-phosphate, *MMP-1*, *MMP-2*, *MMP-3*, *MMP-9*, *MT1-MMP (MMP-14*) tissue remodeling matrix metalloproteinases), *TIMPs*: *TIMP-1*, *TIMP-2*, *TIMP-4* tissue inhibitor of metalloproteinases, *IL1-β* interleukin-1β, *PAF* platelet-activating factor, *LPA* lysophosphatidic acid, *IgG* immunoglobulin G, *Gas6* growth arrest-specific 6, *HMWK* high-molecular-weight kininogen, *ATP* adenosine triphosphate, *GTP* guanosine-5′-triphosphate, *ADP* adenosine diphosphate, *GDP* guanosine diphosphate, *5-HT* serotonin, *TFPI* tissue factor pathway inhibitor

## Thrombocytosis in malignancy

Thrombocytosis in cancer patients is a common finding. However, mechanisms underlying this phenomenon are not fully understood. A variety of tumor-related humoral factors and cytokines influences thrombopoiesis in cancer. Among them are granulocyte colony-stimulating factor (G-CSF), granulocyte macrophage colony-stimulating factor (GM-CSF), interleukin-6 (IL-6), interleukin-1 (IL-1), and thrombopoietin (TPO) [58–63]. Elevated serum levels of TPO were observed in cancer patients with reactive thrombocytosis [64]. It was documented that several tumor cell types can produce and regulate TPO, a key cytokine that stimulates megakaryocyte formation and platelet production, e.g., in ovarian cancer [65, 66]. Recently, experiments performed with orthotopic mouse models of ovarian cancer demonstrated that tumor cell-derived IL-6 stimulates hepatic production of TPO [65]. Activated platelets are a rich source of microparticles (PMPs) that stimulate proliferation, survival, adhesion, and chemotaxis of hematopoietic cells [67]. There exists reciprocal interaction among megakaryocytes and ECs in the bone marrow [62, 68, 69]. Furthermore, it is now known that bone marrow endothelial cells (BMECs) support megakaryocytopoiesis [62]. Various cytokines, such as kit ligand, IL-6, and TPO are constitutively released by BMECs [62]. It was documented that BMECs support proliferation and differentiation of megakaryocytic progenitor cells *in vitro* as well as facilitate the growth and maturation of megakaryocytes *in vivo* [62]. Megakaryocytes release not only cytokines, including IL-1, IL-3, IL-6, and GM-CSF, but also factors essential for the stimulation of angiogenesis, such as VEGF and basic fibroblast growth factor (bFGF) [41, 69, 70]. Hypercoagulability and excessive thrombin generation, commonly observed in the course of malignancy, may contribute to thrombocytosis in cancer patients. In this context, it is of interest that bFGF induces megakaryocyte differentiation through modulation of megakaryocyte-stromal interactions and augmentation of cytokine secretion from megakaryocytes [71]. Moreover, bFGF increases megakaryocyte colony formation *in vitro* [72]. In turn, VEGF contributes to megakaryocyte maturation through an autocrine loop *via* the VEGF receptor—VEGFR-1. Permeability induced by VEGF may be of particular importance since it facilitates megakaryocyte transendothelial migration to the circulation [73]. Platelet infusion promotes bone marrow-derived cell mobilization into the circulation in an ischemic limb model, whereas platelet depletion inhibits the effects [74]. It should be emphasized that tumor-derived proangiogenic factors may facilitate the formation of megakaryocytes that is followed by increased platelet count. Reportedly, platelet count correlates with serum level of VEGF, but not with bFGF in patients with advanced cancer [75]. In turn, angiogenesis inhibitors, such as thrombospondin-1 (TSP-1) and platelet factor 4 (PF-4), inhibit megakaryocytopoiesis in experimental models [76, 77].

## Cancer-associated platelet activation

Increased circulating levels of a platelet-specific α-granule protein, β-thromboglobulin, as well as elevated expression of platelet adhesion molecules (e.g., CD62, CD63, P-selectin) reflect platelet activation. In many cancers (e.g., prostate, breast, lung, gastric, and colon cancer), particularly at an advanced stage of the disease, the β-thromboglobulin levels were significantly elevated [33, 78, 79]. Moreover, increased expression of platelet adhesion receptors was repeatedly reported in blood of cancer patients [80–82].

Adherence of platelets to ECs or ECM components is a prerequisite of local platelet activation and secretion. Of interest then is the mechanism of increased platelet tethering and adhesion in tumor vessels or in tumor-like environments [41]. Platelet adhesion to ECs is increased 2.5-fold after stimulation with VEGF [41]. Tumor-derived ECs are phenotypically and functionally distinct from ECs in normal tissues [83]. Tumor cells induce platelet activation and subsequent aggregation through direct and indirect mechanisms [24, 25]. Both tumors—released soluble stimulators and tumor cell surface molecules—contribute to cancer-related platelet activation [24, 25]. Platelet aggregation in response to tumor cell stimulation is known as tumor cell-induced platelet aggregation (TCIPA) [25]. Indirect platelet activation by cancer cells is through activation of coagulation, which is mainly triggered in malignant disease by tissue factor (TF) present on cancer cells, tumor-infiltrating macrophages, and tumor ECs [33, 34, 84]. Thrombin generated in the process plays a main role among soluble stimulators of TCIPA. Cancer cells also have the ability to release procoagulant microparticles that also initiate thrombin generation [84]. Tumor-derived ADP, cathepsin B, and matrix metalloproteinases also play an important role in TCIPA [85, 86]. Tumor cells produce platelet agonists that mediate tumor cell-platelet interactions and influence the adhesion potential of both types of cells. Increased expression of adhesion molecules on platelet surfaces further intensifies the release of second mediators, which recruit more platelets to the site of interaction [25]. Adhesive properties of tumor cells themselves are responsible for direct interactions between platelets and tumor cells [78]. Likewise, integrins GPIIb-IIIa and GPIb present on platelet membranes play an important role in adhesion reactions with tumor cells in the process of platelet aggregation [79]. Sialoglycoprotein Aggrus/podoplanin present in various tumor cell lines (e.g., glioblastoma, mesothelioma, lung, esophageal squamous cell carcinoma, and colon carcinoma) may also be involved in TCIPA [87, 88]. Namely, the C-type lectin-like receptor (CLEC2) expressed on platelets is a counter receptor of podoplanin. Binding of podoplanin to CLEC-2 transmits platelet activation signals *via* Src family kinases, Syk, and phospholipase Cγ2 in platelets [89, 90]. Furthermore, physical binding of tumor cells and tumor cell-derived plasma membrane vesicles with platelets induces platelet aggregation. Platelets can be activated *via* cancer-induced formation of neutrophil extracellular DNA traps (NETs), that consequently leads to platelet aggregation and thrombus formation [91]. It should be emphasized that tumor cell-induced blood coagulation reinforces aggregation and platelet-mediated tumor cell adhesion to the ECs [92].

## Multidirectional role of platelets in angiogenesis in malignancy

**Fig. 1:**
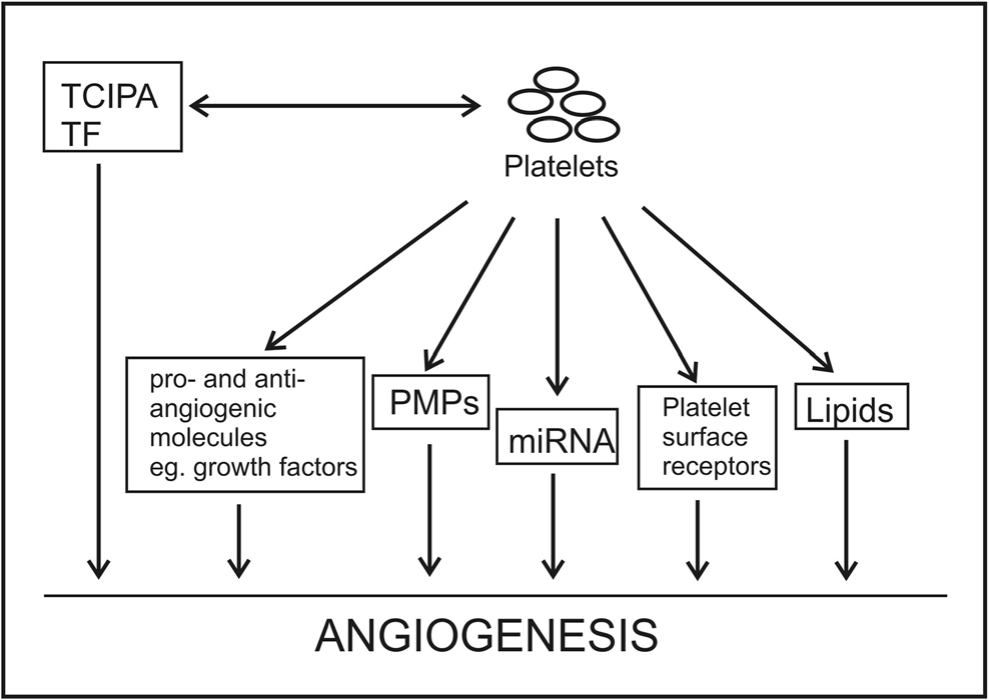
Multidirectional influence of platelets on angiogenesis in malignancy. *TCIPA* tumor cell-induced platelet aggregation, *TF* tissue factor, *PMPs* platelet microparticles, *miRNA* microRNA

Active proliferation of tumor cells necessitates neovascularization to support optimal blood supply for growing tumor tissue in order to deliver necessary nutrients and oxygen, as well as remove waste products from the tumor microenvironment [28–30]. Through numerous studies, it is now apparent that angiogenesis is not only driven by tumor-derived proangiogenic factors but also by tumor microenvironment, stromal cells, and tumor-associated macrophages. Furthermore, reciprocal interplay between tumor cells and ECs contributes to the formation of new blood vessels. The hypothesis that platelets are involved in the process of angiogenesis was raised almost 20 years ago by Pinedo et al. [93]. The presence of activated platelets was observed in the tumor vasculature in sarcoma patients [94]. Platelets were implicated in early and advanced stages of angiogenesis, e.g., in the stabilization of newly formed vessels [39, 42, 43]. Platelets stimulate EC proliferation and tube formation *in vitro* and induce angiogenesis *in vivo* [40, 42, 94], which is dependent on platelet adherence to the differentiating ECs through their surface adhesion molecules [40, 42, 94]. Activated platelets induce TF expression on ECs by interaction between platelet CD154 and CD40 present on ECs to induce coagulation [95]. Ligation of CD40 stimulates expression of adhesion molecules, e.g., E-selectin, vascular cell adhesion molecule-1 (VCAM-1), and intercellular adhesion molecule-1 (ICAM-1) that enhances adhesion of inflammatory cells to the ECs [96]. As platelets adhere almost immediately to exposed or activated endothelium, and they are major storage and delivery vehicles for proangiogenic and antiangiogenic growth factors, bioactive lipids, cytokines, and chemokines, such as stromal-derived factor 1 (SDF-1), platelets orchestrate the local angiogenic stimulus within a tissue and direct the recruitment and differentiation of circulating bone marrow-derived cells (BMDCs) (Fig. 1) [97]. It was documented that platelets are required for BMDC recruitment into ischemia-induced vasculature [74]. Secretion of platelet α-granules, but neither dense granules nor platelet aggregation, is crucial for BMDC homing and subsequent angiogenesis [74, 98]. Furthermore, platelets absorb and sequester tumor-derived proangiogenic factors and induce BMDC mobilization, which is counterbalanced by the antiangiogenic factor TSP-1. A lack of TSP-1 in platelets leads to an imbalance in proangiogenic and antiangiogenic factors and accelerates tumor growth and vascularization [74]. Platelets were demonstrated to stimulate BMDC homing in a VAMP-8-dependent manner [74]. Interestingly, it was revealed that human platelets take up cytokines released by luminal breast cancer cells and thereafter deliver them to indolent metastatic tumor foci, contributing to tumor growth *via* stimulation of vessel formation [99]. Recently, platelets have served as a form of surrogate biosensor as tumor-educated platelets have been used to screen for the presence or absence of cancer (96% accuracy), as well as the type of malignancy (71% accuracy) based on RNA content [100].

## Platelet releasate, receptors, and membrane constituents

Platelet α-granules contain, as mentioned above, proangiogenic and antiangiogenic factors [33]. What particularly links platelets with the process of angiogenesis is that platelet progenitor cells synthesize and release VEGF, while platelets transport and, upon activation, secrete VEGF, which is the most important proangiogenic molecule [38, 39, 41, 75]. The addition of thrombin into platelet-rich plasma from healthy subjects results in much higher VEGF levels than if thrombin is added into platelet-free plasma [101]. Inflammation, a process known to facilitate cancer progression, induces VEGF release from platelets in breast cancer patients [102]. Moreover, the platelet function is altered in cancer patients, as platelets from women with early breast cancer released significantly more VEGF upon thrombin or TF stimulation than platelets derived from healthy controls [103]. In breast, colorectal, renal, and ovarian cancer patients, the platelet count correlated significantly with serum VEGF content [75, 104]. Furthermore, the concentration of platelet-derived VEGF is a better predictor of tumor progression than serum VEGF levels [105]. High serum VEGF concentration per platelet count was associated with shorter overall survival [106]. Of interest is the observation that the ratio between serum VEGF and plasma VEGF tended to be smaller in metastatic breast cancer patients as compared to patients with locoregional disease, suggesting more intense intravascular platelet degranulation in the former group [24]. Cancer cells themselves synthesize VEGF as well as IL-6, that may be released into the bloodstream [107, 108]. Tumor cells *via* IL-6 may exert an indirect effect on thrombocytosis and platelet uptake of free VEGF [108] that may facilitate the transport of VEGF to the site of tumor cell-EC interaction. Tumor cells circulate in the bloodstream in complexes with platelets [25]. Thus, at the sites of tumor cell adherence to ECs, platelets may release their VEGF content to induce permeability, facilitate extravasation of cancer cells, and facilitate angiogenesis at sites of distant metastases.

Tumor cell-derived VEGF may additionally induce the formation of fenestration in the endothelium, a process that leads to the exposure of subendothelial matrix to the circulating blood and consequently induces blood coagulation. Furthermore, VEGF stimulates ECs to secrete vWF, an adhesion molecule for platelets [109]. Irregularity, immaturity of tumor vasculature that is associated with numerous sites of blood flow stasis, and increased interstitial pressure greatly facilitate platelet adhesion to the vessel walls within the tumor. Platelet adhesion and subsequent activation, aggregation, and release promote rolling and adhesion of inflammatory cells on platelet and EC surfaces in the tumor vasculature [110]. All of the abovementioned steps proceed in a self-perpetuating manner to facilitate activation of blood coagulation and subsequent thrombin and fibrin formation.

Serum and platelet VEGF, through induction of vessel permeability, facilitate escape of hemostatic proteins and other macromolecules. It is widely recognized that proteins from the coagulation and fibrinolytic cascades contribute various functions in the process of new vessel formation [111, 112]. Fibrinogen deposited in the extravascular space both at the primary tumor and at metastatic foci forms an ideal surface for platelet binding. It also serves as a basis for EC migration and influences EC motility [32]. Fibrinogen *per se* also activates platelets. VEGF induces TF expression in ECs and tumor-associated monocytes/macrophages [113]. Tissue factor exerts its proangiogenic activity through its signaling function as well as the initiation of TF-dependent blood coagulation, subsequent thrombin generation, and fibrin formation [31, 34]. Interestingly, VEGF induction of platelet adhesion to ECs and platelet activation is dependent on TF activity [94]. Thrombin generated by the TF-dependent pathway of blood coagulation exerts its activity *via* platelet PAR-1 and PAR-4 receptors [111, 112]. Activation of PAR-1 leads to VEGF release from platelets, while activation of PAR-4 leads to the secretion of an inhibitor of angiogenesis, endostatin [114]. Thrombin stimulation results in VEGF/fibronectin complex release from platelets [115]. Binding of VEGF to fibronectin increases VEGF proangiogenic activity (e.g., promoting EC migration) [116].

VEGF supports transendothelial migration of monocytes and serves as a chemotactic factor for monocytes and mast cells [113]. Both tumor-associated monocytes/macrophages and mast cells contribute to angiogenesis in cancer patients [116, 117]. These cells are an additional source of VEGF and other cytokines that indirectly stimulate angiogenesis [113].

Additional proangiogenic factors that are stored in platelet α-granules and released upon platelet activation include VEGF-C (stimulates lymphangiogenesis), bFGF (facilitates migration, proliferation, and differentiation of ECs as well as regulation of the expression of other proangiogenic factors), epidermal growth factor (EGF; upregulates VEGF messenger RNA (mRNA) expression *in vitro* and induces the expression of FGF-binding protein that binds and activates both basic and acidic FGF), platelet-derived endothelial cell growth factor (PD-ECGF; a chemotactic factor for ECs *in vitro* and proangiogenic factor in *in vivo* studies), platelet-derived growth factor (PDGF; mediates angiogenesis *via* stimulation of VEGF expression in ECs localized at the tumor burden and through recruitment of pericytes to newly forming vessels), hepatocyte growth factor (HGF; stimulates the expression of VEGF and HGF itself in ECs as well as the synthesis of platelet activating factor (PAF) by macrophages that, in turn, promotes further platelet activation and increases EC migration), insulin-like growth factor 1 and 2 (IGF-1, IGF-2; mediates proteolytic events necessary in angiogenesis), transforming growth factor-beta 1 (TGF-β1; increases EC survival and stabilizes capillary structures in the *in vitro* models of angiogenesis), angiopoietin-1 (Ang-1), and plasminogen activator inhibitor-1 (PAI-1) [33]. Alpha granules also contain PAI-1. Although it may be considered an angiogenesis inhibitor (diminishing the dynamic of proteolytic events), PAI-1 promotes angiogenesis [118]. In the experimental studies, new vessel formation was abolished in the absence of PAI-1 [119]. Interestingly, VEGF, platelet factor 4 (PF4), and PDGF are elevated in platelets of colorectal cancer patients [120].

Platelet α-granules are a source of antiangiogenic factors as well. PF-4 bound to the surface of heparin-like glycosaminoglycans on ECs, blocks binding sites for heparin-binding endothelial growth factors, directly neutralizes the heparin-binding region of bFGF, and inhibits the EC stimulatory activity exerted by EGF and VEGF [33]. Thrombospondin-1 stimulates EC adhesion and spreading but inhibits the chemotactic response of ECs to bFGF. It may facilitate growth factor and integrin signaling pathways between ECs and block fibrin-induced EC motility [33, 121]. Smaller fragments of HGF (NK1—first HGF kringle domain, NK2—first two kringle domains) endowed with antiangiogenic activity are also stored in platelet α-granules. They inhibit angiogenesis induced by HGF in experimental models [33]. Angiopoietin-1, considered an angiogenesis stimulator, in some circumstances may exert an antiangiogenic function as it inhibits endothelial permeability and IL-8 synthesis [33]. Angiogenesis inhibitor, angiostatin, was also identified in human platelets [122]. Platelet membranes constitutively generate angiostatin, the mechanism of which is dependent on uPA but not MMPs [123]. Endostatin is also in α-granules [124]. Activation of PAR-4 contributes to the secretion of this angiogenesis inhibitor, while activation of PAR-1 leads to the suppression of endostatin release from platelets [124]. Furthermore, platelet membranes have receptors for growth factors [121, 125]. PDGF receptors are present on platelets as well as on their progenitor cells, megakaryocytes, where they function to enhance their proliferation rate [126]. Platelet PDGF-α receptors mediate negative feedback regulation [125].

Platelet membranes also have receptors for VEGF (both VEGFR-1 and VEGFR-2) [126, ]. While VEGF does not induce aggregation itself, it facilitates SFRLLN-stimulated or thrombin-stimulated platelet aggregation [127]. It may be suggested that platelets transport the receptors and transfer them to the site of neoangiogenesis through PMP formation.

The other type of platelet granules, dense granules, releases ADP, which was demonstrated to exert chemotactic activity toward ECs, thus facilitating migratory events [128]. However, recently, it was noted that neither dense granule content nor lysosomal secretion is critical for new blood vessel formation [43].

Platelets are surrounded by phospholipid membrane. Three constituents of the membrane possess proangiogenic activity, namely phosphatidic acid, lysophosphatidate, and S1P. S1P, a bioactive lipid released by activated platelets during blood clotting, is a potent EC chemoattractant [129]. It exerts its effect in a receptor-dependent process [129].

## Platelet-derived microparticles

Activated platelets form microvesicles that are released into the blood [130]. Platelet remnants and microvesicles were found at sites of angiogenic sprouting [131]. Platelet-derived microparticles, known also as microvesicles, are the most abundant microparticle constituents in the peripheral blood, accounting for around 70–90% of all extracellular vesicles [132]. They are formed by cell membrane budding, and their size varies between 0.1 and 1 μm [133]. Platelet-derived microparticles are constantly shed into the circulation at certain levels even in healthy people [134]. Some fraction of circulating PMPs originates from megakaryocytes [132]. An increased number of PMPs was observed in thrombotic disorders as well as in solid tumors and hematologic malignancies [132, 135]. Platelet activation, oxidative stress, tissue hypoxia, and activation of the coagulation cascade stimulate production of PMPs [136]. In addition to actively promoting tumor growth and metastatic dissemination, PMPs also promote angiogenesis [132, 134–140]. Platelet-derived microparticle function is multifactorial. They induce sprouting of new blood vessels both *in vitro* and *in vivo* to a degree comparable with that of whole platelets, and they enhance vascular permeability [139, 141]. They induce the procoagulant phenotype through PS-induced activation of blood-born TF or by triggering TF expression in monocytes that contributes to proangiogenic thrombin and fibrin formation [34, 130]. PMP membranes are also enriched with TF and display negatively charged surfaces where clotting factor complexes can assemble. This substantially induces blood coagulation and consequently tumor angiogenesis [31, 33, 34, 142]. They were documented to stimulate the expression of adhesion molecules on a variety of cells, promote the release of cytokines, influence vascular reactivity, and induce inflammation [67, 138]. Platelet-derived microparticles can express and transfer functional receptors from platelet membranes, such as glycoprotein IIb-IIIa (GPIIb-IIIa) and P-selectin, to different cell types [143], and as a consequence facilitate engraftment of hematopoietic stem/progenitor cells [140]. They can transfer angiogenic factors intracellularly and can induce proangiogenic genes through direct cellular contact, e.g., with ECs or fusion with target cells, such as tumor cells or ECs [133]. Interestingly, the presence of PMPs in endothelial progenitor cell cultures was also observed [142, ]. PMPs chemoattract hematopoietic cells and induce their adhesion, survival, and proliferation [67]. Moreover, *in vitro* and *in vivo* studies demonstrated that PMPs promote proliferation and survival of ECs [138]. Furthermore, *ex vivo* studies demonstrated that PMPs stimulate progenitor cells to form a capillary network [139, 142]. The platelet origin of PMPs results in the presence of proangiogenic growth factors (e.g., VEGF, PDGF, FGF) as well as metalloproteases in their α-granules [133]. Moreover, PMPs stimulate secretion of proangiogenic factors by tumor cells [144]. PMPs have the ability to induce the expression of MMP-9, VEGF, and IL-8, all of which are known to be involved in angiogenesis [67]. Furthermore, *in vitro* PMPs stimulate prostate cancer cells to secrete MMP-2, which, in turn, facilitates their passage through collagen, a major component of ECM [145]. Interestingly, this secretion is not mediated by major intraplatelet proangiogenic factors, such as VEGF, bFGF, or platelet factor 4 [145]. PMPs are able to stimulate kinase-dependent protein phoshorylation (MAPK p42/44 and AKT) and increase the expression of membrane metalloproteinase type 1 (MT1-MMP) that degrades components of the extracellular matrix to facilitate angiogenesis [140, 146]. Increased activity of MMPs was documented in many tumor types (e.g., MT1-MMP is overexpressed in lung cancer) [146]. Recently, it was reported that PMPs transfer proteins as well as DNA and RNA (including mRNA and miRNA) to recipient vascular cells and other cell types [147–149].

## MicroRNA

MicroRNA (miRNA/miRs) are small non-coding RNAs, approximately 18–25 nucleotides in length that are able to modulate post-transcriptional regulation of gene expression and function of protein-coding mRNAs in almost all key cellular processes, including, among others, angiogenesis, cell proliferation, migration, and apoptosis [150]. miRNA is transcribed in the nucleus by RNA polymerase II as a primary transcript called pri-miRNA [151]. It is recognized further by Drosha ribonuclease and its partner, the double-stranded RNA binding protein DGCR8 [152] that go on to generate precursor miRNA (pre-miRNA) of approximately 70 nucleotides [151]. The latter is then exported from the nucleus to the cytoplasm by exportin 5 (XPO5) [153] and cleaved by RNase III enzyme Dicer, RNA-binding protein 2 (TARBP2), and AGO2 (DICER complex). The processing produces a double-stranded miRNA-miRNA duplex* [154]. After separation of the two strands, the mature miRNA (the guide strand) is incorporated into the RNA-induced silencing complex (RISC), while the passenger miRNA strand denoted as * is incorporated into the RISC complex or degraded [155]. The mature miRNA guides the AGO protein of the RISC to the complementary mRNA sequence on the target to repress its expression [151]. The six to eight nucleotide sequence at the 5′ end of the loaded miRNA binds to the complementary sequence on the mRNA inducing their translational repression or degradation. Each miRNA is capable of regulating the expression of many genes; thus, each miRNA can simultaneously regulate a variety of cellular signaling pathways. Human platelets contain an abundant and diverse repertoire of miRNAs [156, 157] that may regulate platelet mRNAs, protein synthesis, and reactivity [157–159]. Platelets can release miRNAs directly into circulation as vesicle-free ribonucleoprotein complexes in association with Ago2 or high-density lipoproteins (HDL), or in exosomes, shedding vesicles, apoptotic bodies, and PMPs [160–163]. Since platelets release PMPs upon activation, and PMPs are the most abundant microvesicles in the circulation, they carry a substantial amount of miRNAs that potentially control angiogenesis [148, 157]. miRNA, e.g., miR-19, miR-21, miR-126, miR-133, miR-146, miR-223, has been detected in PMPs [149]. Delivery of functional platelet miRNAs into ECs *via* PMPs has also been demonstrated [165, 166], where activated platelets released functional miRNAs that entered into ECs to regulate endothelial ICAM-1 expression [165]. Furthermore, Laffont et al. [166], in an elegant study, documented that platelets activated with thrombin release miR-223 preferentially through PMPs that can be internalized by ECs (human umbilical endothelial cells (HUVECs)), leading to the accumulation of platelet-derived miR-223. They also demonstrated that PMPs contain functional Ago2 × miR-223 complexes that are able to regulate (downregulate) expression of endogenous genes in recipient HUVECs, both at the mRNA (mRNA destabilization) and protein (inhibition of mRNA translation initiation) levels [166]. Platelet-released miR-223 promotes advanced glycation end product-induced vascular EC apoptosis by targeting insulin-like growth factor 1 receptor [167]. Platelet-derived miR-223 regulates P2Y_12_ receptor expression in platelets, suggesting accelerated platelet activation and aggregation that may contribute to further stimulation of angiogenesis [168]. Vascular endothelium damage increases the level of apoptotic bodies that induce the expression of SDH-1 in recipient ECs through importing miR-126 [163]. Additionally, miR-126 targets the protein regulator of G-protein signaling 16 (RGS16) that is known to inhibit CXCR4 [163]. Consequently, this enables CXCR4 to stimulate an autoregulatory feedback loop that increases the phosphorylation of ERK1/2 and enhances the production of SDF-1 [163]. Furthermore, introduction of apoptotic bodies into an animal model by injection into the blood stream results in elevated levels of miR-126, and subsequent dysfunction of endothelium [168]. It was demonstrated that miR-126 enhances vascular hemostasis by protecting endothelial integrity as it targets SPRED1 and PIK2R2 (inhibitors of EC growth signaling) [169]. Platelet-derived miR-140 directly targets SDF-1 in fibroblasts, which may also contribute to angiogenesis [170]. Furthermore, miR-221 and miR-222 target c-kit (tyrosine-protein kinase kit), endothelial nitric oxide synthase (eNOS), and p27/lip1 subsequently promote angiogenesis *in vivo* in response to stem cell factor [171, 172]. Given the immense diversity of platelet miRNA sequences [157] and the number of cell types capable of exchanging information by intercellular transfer [164], one quickly appreciates the complexity of intercellular communication. Though miRNAs may be critically involved in angiogenesis, their role in platelet secretion and platelet-mediated angiogenesis has not been fully elucidated. The net influence of miRNA and PMP-derived miRNA on angiogenesis warrants further study.

## Net balance of platelet proangiogenic/antiangiogenic activity

As previously described, platelets are the source of both stimulators and inhibitors of angiogenesis. Both proangiogenic and antiangiogenic factors are stored in distinct α-granules [173, 174], and their release is induced by a selective stimulation of PAR-1 and PAR-4 receptors [173]. Namely, PAR-1 activation leads to VEGF release, whereas stimulation of PAR-4 results in the secretion of antiangiogenic endostatin [173]. Of interest, tumor cell-derived ADP through activation of P2Y_12_ receptor induces release of VEGF, but does not affect the secretion of endostatin [175, 176]. In contrast, thromboxane A2 was documented to facilitate endostatin release, but not VEGF secretion [175]. As platelet activation leads to simultaneous stimulation of antagonistic pathways, it is unclear whether such a subtle mechanism of regulation to selectively release proangiogenic and antiangiogenic factors occurs *in vivo*. Recently, experiments performed with immunofluorescence microscopy and micro ELISA assays revealed contradictory results [177, 178]. Nevertheless, it should be emphasized that the absence of platelets inhibits the early stages of angiogenesis and results in fewer new vessels *in vivo* [43, 129]. Platelet releasate stimulates EC migration, and the addition of platelets into the Matrigel model before injection induces angiogenesis [139]. Gastric ulcer healing, that is dependent on angiogenesis, is also inhibited in the presence of thrombocytopenia [114]. Recently, the important role of PMPs and miRNA in the promotion of angiogenesis was documented [33, 133, 138, 139]. Platelets also play an important role in the stabilization of newly formed vessels [43]. All of the above data indicate support in the overall stimulatory effect of platelets on tumor angiogenesis. It raises the possibility that interfering with platelet function may be an effective antineoplastic treatment strategy [179, 180]. Many preclinical and clinical studies dedicated to this idea are ongoing.
